# Prognostic value and underlying mechanism of autophagy-related genes in bladder cancer

**DOI:** 10.1038/s41598-022-06334-0

**Published:** 2022-02-09

**Authors:** Shiyuan Peng, Shanjin Ma, Fa Yang, Chao Xu, Hongji Li, Shiqi Lu, Jingliang Zhang, Jianhua Jiao, Donghui Han, Changhong Shi, Rui Zhang, An-Gang Yang, Keying Zhang, Weihong Wen, Weijun Qin

**Affiliations:** 1 Department of Urology, Xijing Hospital, Fourth Military Medical University, Xi’an, 710032 China; 2 Department of Urology, Tangdu Hospital, Fourth Military Medical University, Xi’an, 710032 China; 3 Institute of Medical Research, Northwestern Polytechnical University, Xi’an, 710072 China; 4 Laboratory Animal Center, Fourth Military Medical University, Xi’an, 710032 China; 5 State Key Laboratory of Cancer Biology, Department of Immunology, Fourth Military Medical University, Xi’an, 710032 China

**Keywords:** Cancer, Urology

## Abstract

Bladder cancer (BLCA) is the most common malignancy whose early diagnosis can ensure a better prognosis. However, the predictive accuracy of commonly used predictors, including patients’ general condition, histological grade, and pathological stage, is insufficient to identify the patients who need invasive treatment. Autophagy is regarded as a vital factor in maintaining mitochondrial function and energy homeostasis in cancer cells. Whether autophagy-related genes (ARGs) can predict the prognosis of BLCA patients deserves to be investigated. Based on BLCA data retrieved from the Cancer Genome Atlas and ARGs list obtained from the Human Autophagy Database website, we identified prognosis-related differentially expressed ARGs (PDEARGs) through Wilcox text and constructed a PDEARGs-based prognostic model through multivariate Cox regression analysis. The predictive accuracy, independent forecasting capability, and the correlation between present model and clinical variables or tumor microenvironment were evaluated through R software. Enrichment analysis of PDEARGs was performed to explore the underlying mechanism, and a systematic prognostic signature with nomogram was constructed by integrating clinical variables and the aforementioned PDEARGs-based model. We found that the risk score generated by PDEARGs-based model could effectively reflect deteriorated clinical variables and tumor-promoting microenvironment. Additionally, several immune-related gene ontology terms were significantly enriched by PDEARGs, which might provide insights for present model and propose potential therapeutic targets for BLCA patients. Finally, a systematic prognostic signature with promoted clinical utility and predictive accuracy was constructed to assist clinician decision. PDEARGs are valuable prognostic predictors and potential therapeutic targets for BLCA patients.

## Introduction

Bladder cancer (BLCA) is the most common malignancy of the urinary system, with about 549,000 new cases and 200,000 death worldwide in 2018^1,2^. BLCA is characterized by a high rate of recurrence and progression, which impose a considerable economic burden on the healthcare system and have substantial effects on the quality of life and overall outcome of BLCA patients. Although novel treatments for BLCA have been proposed, such as immunotherapy of PD-1/PD-L1^3^, the 5-year survival rate for advanced/metastatic BLCA is only 15%, and the median overall survival is less than 15 months^4^. Early diagnosis and recurrence monitoring of BLCA would be valuable for a better prognosis. However, the predictive accuracy of commonly used predictors, including patients’ general condition, histological grade, and pathological stage, is insufficient to identify the patients who need invasive treatment^5^. Recently, the biomarker-based signature is regarded as a promising tool to predict the prognosis of BLCA patients and assist clinical decisions^6^.

Autophagy is a highly conserved cellular self-degradative process. Some cancers could use autophagy-mediated recycling to maintain mitochondrial function and energy homeostasis to meet their elevated metabolic demand for growth and proliferation^7^. Therefore, autophagy-based prognostic signature has been widely investigated, and autophagy inhibition has been proposed as a novel cancer therapy strategy^8^. As reported, autophagy-related genes (ARGs) can effectively select high-risk colorectal cancer patients who require more aggressive therapeutic interventions^9^. Liu et al. constructed a 22-ARGs-based signature that could independently predict the overall survival (OS) in TCGA lung adenocarcinoma^10^. ARGs-based signature's predictive value has also been validated in glioblastoma^11^ and breast cancer^12^. However, the clinical relevance and prognostic significance of ARGs-based signature in BLCA remain unknown.

In present study, we aimed to develop a reliable prognostic model for BLCA patients using multiple ARGs and investigate its clinical implications. Furthermore, we evaluated the correlation between present prognostic model and tumor microenvironment (TME) and explored the underlying mechanism. Finally, to facilitate clinical utility of the model mentioned above, a systematic prognostic signature was constructed by integrating clinical predictors and ARGs-based molecular biomarkers.

## Materials and methods

### Data source and preprocessing

**Figure 1:**
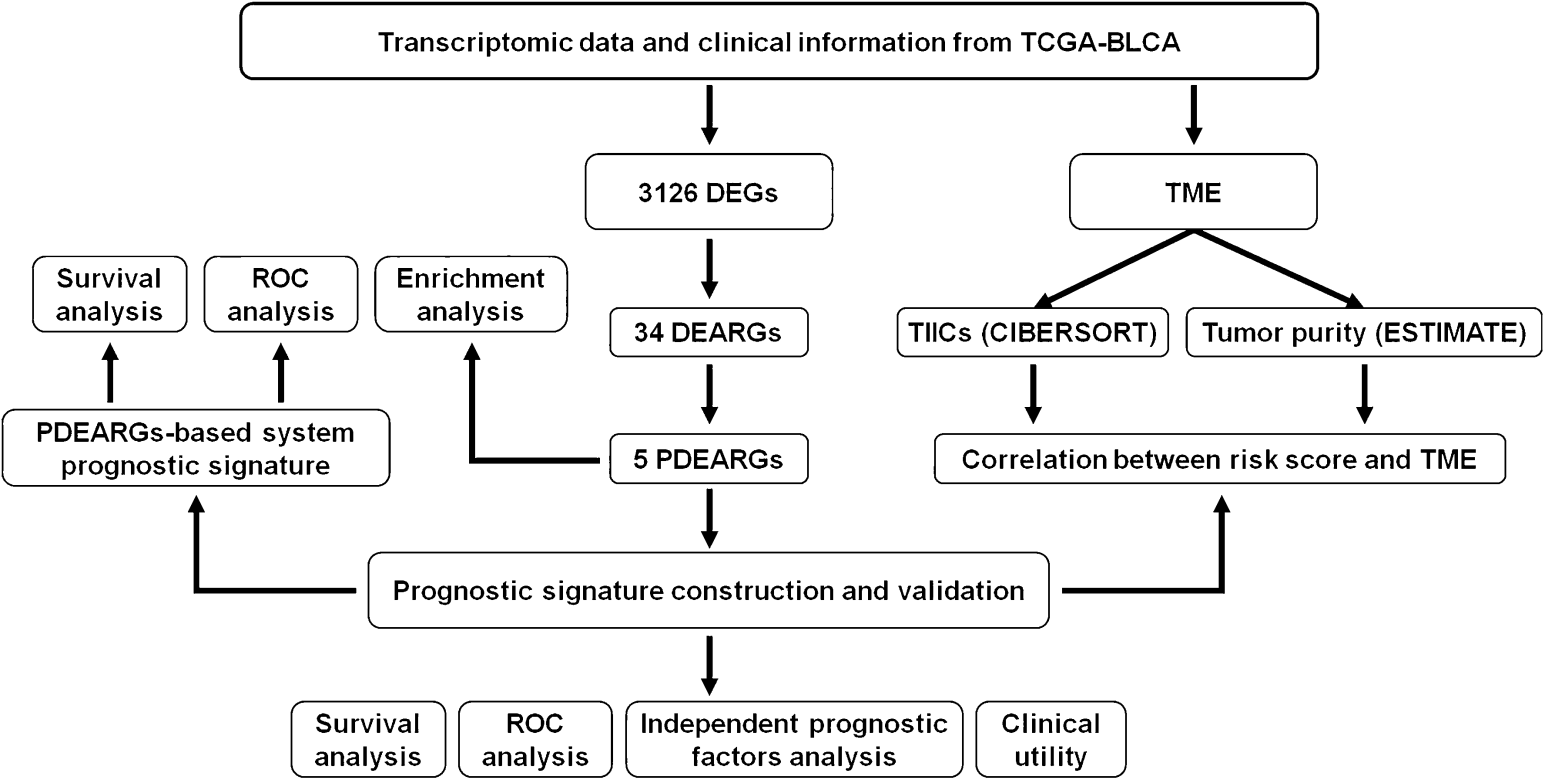
Flow diagram of the present study.

Transcriptomic data (RNA-Seq FPKM) and clinical information were downloaded from The Cancer Genome Atlas (TCGA) portal (https://portal.gdc.cancer.gov/), including 413 BLCA data and 19 non-tumor data. After integrating those data through ID numbers, the gene measured with multi-probes were replaced with their average via limma package (http://www.bioconductor.org/packages/release/bioc/html/limma.html)^13^. Then, 371 patients with follow-up time > 90 d and complete data were selected for further analyses. All data were processed and analyzed with R software 3.6.0 (https://www.r-project.org/), and the flow diagram of present study was shown in Fig. 1.

### Differential expression analysis and ARGs identification

DEGs between tumor tissues and normal tissues were analyzed through Wilcoxon test. p-value was adjusted with FDR, and filter criteria was FDR < 0.05 and|log_2_ fold-change [FC]|> 1. DEARGs were identified by matching the DEGs with the latest list of ARGs obtained from the Human Autophagy Database (HADb) website (http://www.autophagy.lu/index.html). Univariate Cox regression analysis was used to identify possible PDEARGs (*P* < 0.05).

### Prognostic signature construction and validation

Survival R package (https://cran.r-project.org/web/packages/survival/index.html) was adopted to construct a PDEARGs-based prognostic model. To avoid overfitting, PDEARGs that correlated highly with other genes were deleted. Then, Cox proportional hazards regression was used to build a prognostic risk model, and the regression coefficients were used to weight the expression value of selected PDEARGs. The risk score of each patient was calculated using the following formula^14^:

$${\text{Risk}}\;{\text{score}} = \mathop \sum \limits_{i = 1}^{n} \;{\text{coefficient}} \left( {{\text{gene}}\;{\text{i}}} \right)*{\text{Expression}}\;{\text{value}}\;{\text{of}}\left( {{\text{gene}}\;{\text{i}}} \right)$$

Individuals were separated into high-risk or low-risk groups according to the median risk score. Subsequently, survival analysis and receiver operating characteristic (ROC) analysis were performed as reported^6^. The expression level of the five selected PDEARGs between two risk groups was analyzed with R software 3.6.0.

### Quantitative real-time PCR (qRT-PCR) analysis

The expression level of five selected PDEARGs between tumor and adjacent tissues were further detected through qRT-PCR. Briefly, the BLCA samples used for present assay were collected from three BLCA patients who underwent radical cystectomy at Xijing Hospital, and corresponding paracancerous tissue were used as control. The clinical characterization of selected patients was shown in Supplementary Table 1. Then, TRIzol™ Reagent (Cat#15596018, Invitrogen, USA) was used to isolate the total RNA according to the manufacturer’s protocol. The RevertAid First Strand cDNA Synthesis Kit (Cat#RR036A, TAKARA, Japan) was used to synthesize cDNA from 500 ng of total RNA. qRT-PCR was carried out by using TB Green Premix Ex Taq II Kit (Cat#RR820A, TAKARA, Japan) on a BioRad CFX96 system. GAPDH served as an endogenous control. The sequences of the primers are listed in Supporting Data 1. All experiments reported in this study were conducted according to an experimental protocol approved by the Research Ethics Committee of the Fourth Military Medical University. All methods were performed in accordance with the relevant guidelines and regulations, and all of the participating patients gave their informed written consent.

### Independent prognostic factors analysis

Univariate Cox regression analysis was performed to identify factors affecting the OS of BLCA patients. Then, multivariate Cox regression analysis was used to evaluate whether the risk score generated from the present prognostic model could be used as an independent prognostic factor. *P* < 0.05 was considered statistically significant.

### Clinical utility of prognostic signature

The relationship between risk factors of the present model and certain clinical variables (i.e., age, gender, histological grade, pathological stage, and tumor-node-metastasis (TNM) status) was analyzed with *t*-test. The box plot was prepared with beeswarm R package (https://cran.r-project.org/web/packages/beeswarm/index.html), and the impact of PDEARGs on BLCA prognosis was analyzed through survival R package (https://cran.r-project.org/web/packages/survivalAnalysis/index.html).

### Correlation between risk score and TME

Tumor purity, infiltrating stromal and immune cells of TCGA-BLCA were assessed through ESTIMATE R package (https://r-forge.r-project.org/projects/estimate/) as previously reported^15^. The relative fraction of 22 TIICs types in each sample was quantified by the CIBERSORT method and LM22 signature matrix (https://rdrr.io/github/singha53/amritr/src/R/supportFunc_cibersort.R)^16,17^. The algorithm ran at 100 permutations with a threshold of *P* < 0.05 to select eligible patients for further analysis^18^. The correlation between risk score and TME was analyzed with the Pearson correlation coefficient test, and the impact of TME on clinical variables was evaluated as well.

### Enrichment analysis of PDEARGs

GO function enrichment of the five selected PDEARGs was performed via clusterProfiler and enrichplot R package (http://master.bioconductor.org/). FDR < 0.05 was considered statistically significant. Then, the top 10 terms involved with different GO functions were plotted. To better explain the relationship between PDEARGs and GO function, a chord plot of representative terms was constructed with Goplot R package (http://wencke.github.io/).

### Construction and validation of PDEARGs-based systematic prognostic signature

A systematic prognostic signature was constructed through a similar method mentioned above by integrating seven clinical variables (i.e., age, gender, histological grade, pathological stage, and TNM status) with the signature discussed above. The variables highly correlated with others were deleted to avoid overfitting, and the regression coefficients were used to weight selected variables. The median of the systematic risk was used to separate the patients into two risk groups, and survival probability was analyzed using R software 3.6.0. Predictive accuracy of the novel systematic signature was evaluated with survivalROC R package (https://cran.r-project.org/web/packages/survivalROC/index.html). Finally, the systematic signature was visualized through a nomogram constructed by rms R package (https://cran.r-project.org/web/packages/rms/index.html).

### External validation of both prognostic signature

GSE13507 and GSE31684 cohorts with gene expression data and clinical information were downloaded from gene expression omnibus (GEO) database (https://www.ncbi.nlm.nih.gov/geo/). Patients with follow-up time < 90 d or other incomplete data were removed. Then, the risk score and systematic risk of 143 patients were calculated. The efficacy of present signatures was validated in external datasets with survival analysis. The prognostic value of selected PDEARGs was estimated as well.

## Results

### Identification of BLCA specific PDEARGs

**Figure 2:**
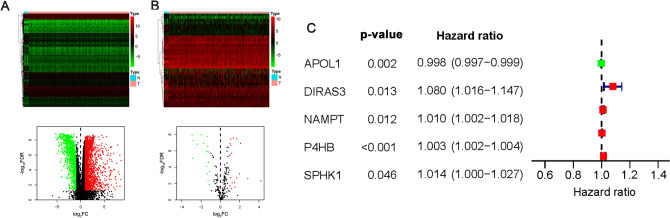
Identification of BLCA specific DEGs, DEARGs, and PDEARGs. (**A**) Heat map and volcano plot of DEGs. (**B**) Heat map and volcano plot of DEARGs. The green to red spectrum indicates low to high gene expression in the heat map; the red, green and black dots represent upregulated, downregulated and unchanged genes in the volcano plot, respectively. (**C**) Forest graph of PDEARGs. The red and green dots represent PDEARGs with a hazard ratio > 1 and ≤ 1, respectively.

We obtained 3126 differentially expressed genes (DEGs) based on the TCGA-BLCA dataset, among which 1223 genes were downregulated, and 1903 genes were upregulated in tumor tissues compared with normal tissues (false discovery rate (FDR) < 0.05, |log_2_ FC|> 1; Fig. 2A). Then, 34 BLCA-specific differentially expressed ARGs (DEARGs) were identified (Fig. 2B), and five prognostic DEARGs (PDEARGs) were found to be significantly associated with the OS of BLCA patients (*P* < 0.05; Fig. 2C). Among the five PDEARGs, *APOL1* (apolipoprotein L1) with hazard ratio ≤ one was regarded as a protective gene, while the other four genes [*DIRAS3* (DIRAS family, GTP-binding RAS-like 3), *NAMPT* (Nicotinamide phosphoribosyltransferase), *P4HB* (Proly 4-hydroxylase beta polypeptide), and *SPHK1* (Sphingosine kinase-1)] were identified as high-risk genes predicting a poor prognosis of BLCA.

### Construction of PDEARGs-based prognostic signature

The regression coefficients were employed to construct a five PDEARGs-based prognostic risk model (Table 1). Subsequently, the risk score for each patient was calculated with the following computational formula:

$$\begin{aligned} {\text{Risk}}\;{\text{score}} & = \left( { - 0.0019 \times {\text{expression}}\;{\text{of}}\;APOL1} \right) + \left( {0.0775 \times {\text{expression}}\;{\text{of}}\;DIRAS3} \right) \, \\ & \quad + \left( {0.0127 \times {\text{expression}}\;{\text{of}}\;NAMPT} \right) + \left( {0.0030 \times {\text{expression}}\;{\text{of}}\;P4HB} \right) \\ & \quad + \left( {0.0120 \times {\text{expression}}\;{\text{of}}\;SPHK1} \right). \\ \end{aligned}$$

**Table 1:** Construction of five PDEARGs-based prognostic risk model.

Genes	Coefficient	HR	HR.95L	HR.95H	*P* value
*APOL1*	− 0.0019	0.9981	0.9968	0.9995	0.0058
*DIRAS3*	0.0775	1.0806	1.0116	1.1543	0.0212
*NAMPT*	0.0127	1.0128	1.0047	1.0210	0.0019
*P4HB*	0.0030	1.0030	1.0017	1.0043	0.0000
*SPHK1*	0.0120	1.0121	0.9979	1.0264	0.0944

*HR* hazard ratio, *HR.95H and HR.95L* 95% confidence interval.

**Figure 3:**
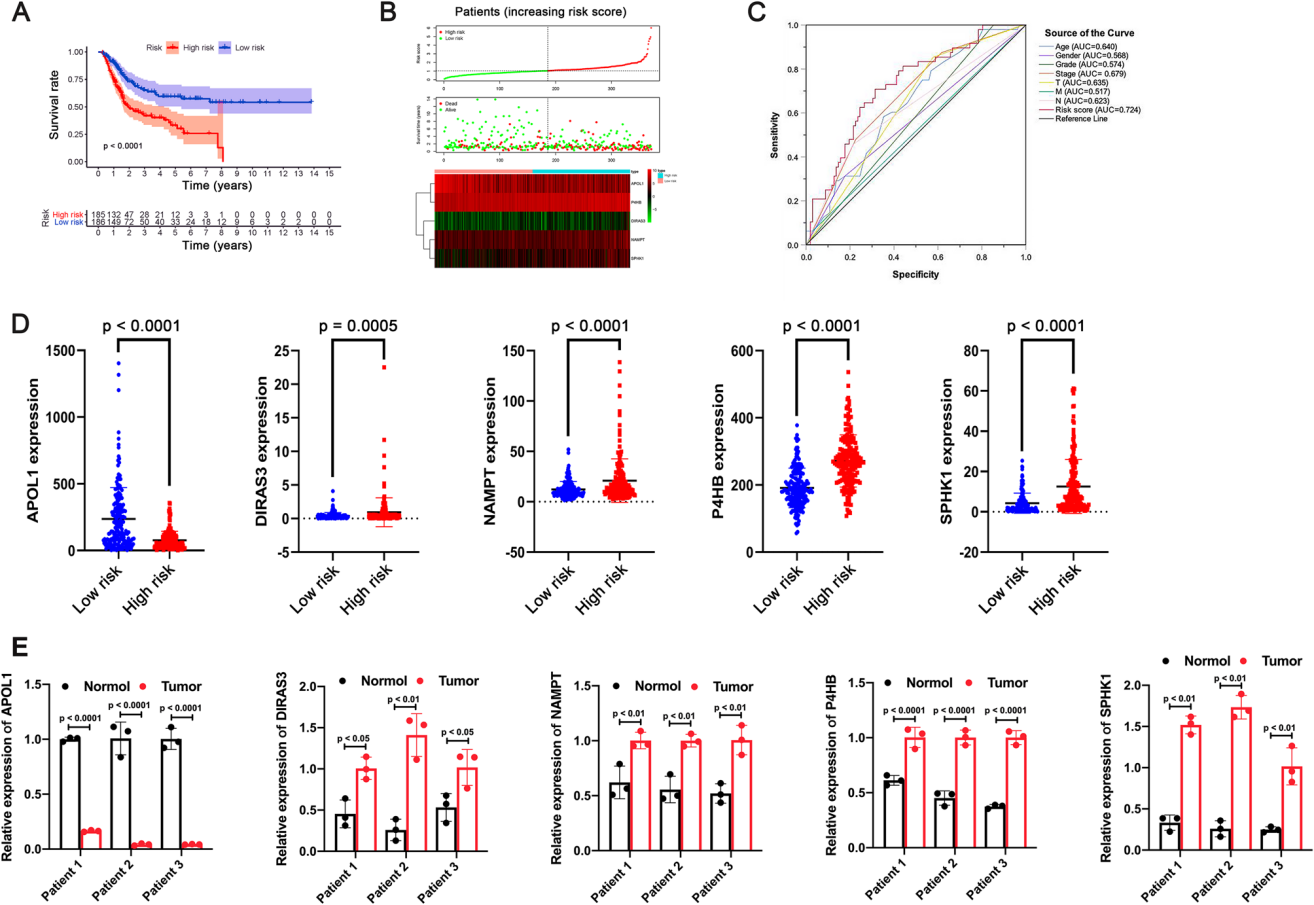
Prognostic value of present risk model. (**A**) Survival analysis between high-risk and low-risk groups. The 95% confidence interval was shown as a light-colored background around the Kaplan–Meier curve. (**B**) Risk plot encompassing the distribution of risk score, survival status, and risk genes expression of each patient. (**C**) ROC curve analysis of different variables. female = 1, male = 0. (**D**) Expression level of the five selected PDEARGs between low-risk and high-risk group. (**E**) Expression level of the five selected PDEARGs between tumor and adjacent normal tissue (n = 3). Data are presented as the means ± SD from 3 independent experiments.

Individuals were sorted into a high-risk group (n = 185) and a low-risk group (n = 186) by the median risk score of 1.02. Survival analysis indicated that the prognosis was poorer in the high-risk group than in the low-risk group (*P* < 0.001; Fig. 3A). Precisely, the 5-year OS rate in the high-risk group was 33.2%, while it was 59.5% in the low-risk group. Then, we analyzed the distribution of each patient's risk score and survival status, and a large amount of death existed in the high-risk group (Fig. 3B). As well, a heat map and a series of box plots were generated to depict the expression level of the five selected PDEARGs, among which the protective gene (*APOL1*) was downregulated, and the other four risk genes were upregulated in patients with high-risk score (Fig. 3B, D). The area under the curve (AUC) was 0.724, which was much higher than other clinical parameters, suggesting that the present model was more accurate in predicting BLCA patients' OS (Fig. 3C).

To validate the RNA-seq data, we analyzed the expression level of five selected PDEARGs using qRT-PCR. As shown in Fig. 3E and Supplementary Table 2, the protective gene (*APOL1*) was downregulated, while the other four risk genes were upregulated in tumor, compared with the adjacent normal tissue, which were consistent with the RNA-seq data above.

### Independent prognostic value of present signature

**Figure 4:**
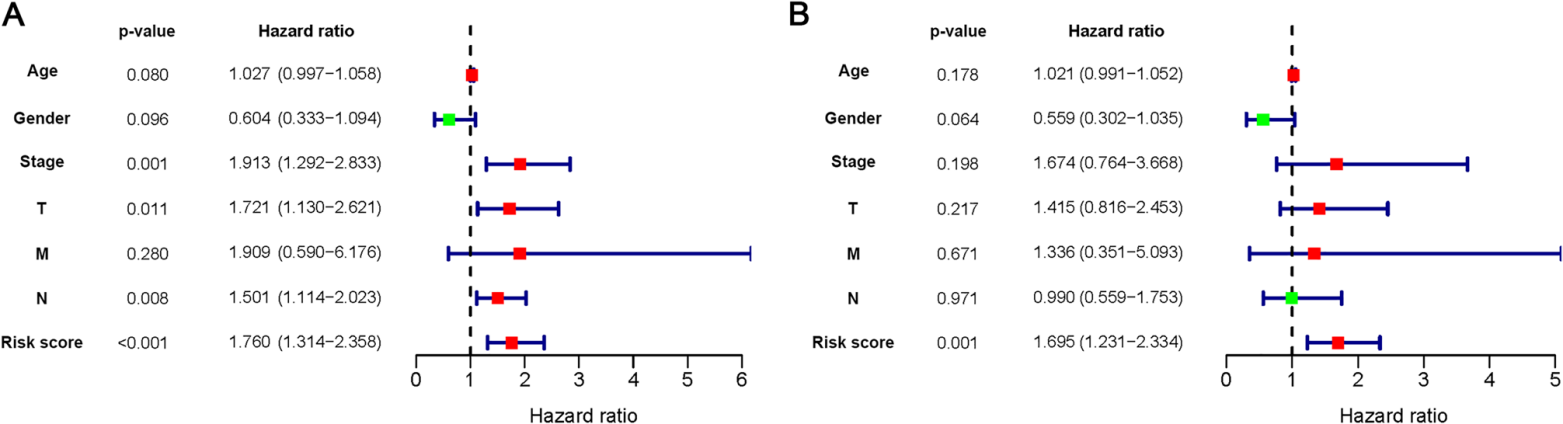
Univariate and multivariate Cox regression analysis. (**A**) Univariate Cox regression analysis to identify prognosis associated factors. (**B**) Multivariate Cox regression analysis to assess independent prognostic factors. The red and green dots represent variables with hazard ratio > 1 and ≤ 1, respectively.

As shown in Fig. 4A, the variables of pathological stage, tumor-node-metastasis (TNM) status, and risk score were associated with the prognosis of BLCA patients (*P* < 0.05). Multivariate analysis showed that the risk score was an independent prognostic factor for OS (*P* < 0.01; Fig. 4B). The hazard ratio for the risk score was 1.695, indicating a high-risk score would predict a bad prognosis. The commonly used clinical variables, such as age, gender, pathological stage, and TNM status, were insufficient to serve as independent prognostic predictors (*P* > 0.05).

### Clinical utility of present signature

**Figure 5:**
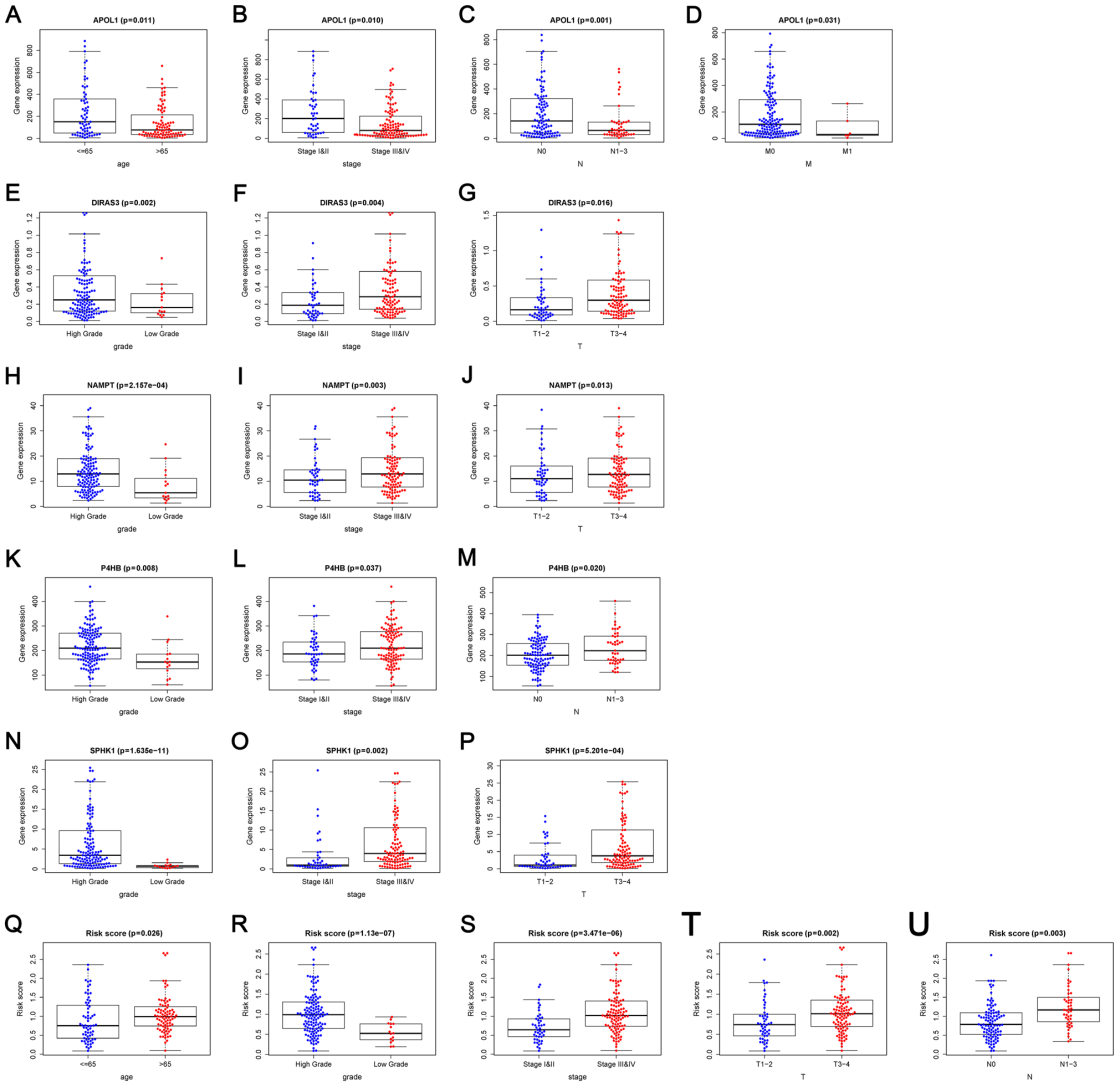
Relationship between prognostic model related factors and clinical variables. (**A**–**D**) *APOL1* and clinical variables. (**E**–**G**) *DIRAS3* and clinical variables. (**H**–**J**) *NAMPT* and clinical variables. (**K**–**M**) *P4HB* and clinical variables. (**N**–**P**) *SPHK1* and clinical variables. (**Q**–**U**) Risk score and clinical variables. Box plots with *P* < 0.05 are shown.

**Figure 6:**
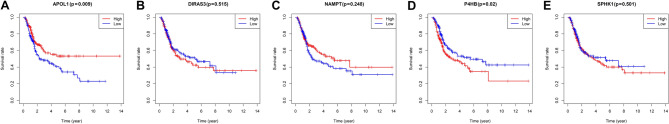
Survival analysis of prognostic model related genes. Kaplan–Meier curve of patients with different expression levels of *APOL1* (**A**), *DIRAS3* (**B**), *NAMPT* (**C**), *P4HB* (**D**), *SPHK1* (**E**). The median expression level was used as the cut-off value. Red line and blue line represent high expression and low expression group, respectively.

The relationship between present model and clinical variables was analyzed (Table 2). High-expression of *APOL1* was associated with decreased pathological stage, N status, and M status (*P* < 0.05; Fig. 5B–D). Contrarily, as the value of risk score or the expression of the other four risk genes (*DIRAS3*, *NAMPT*, *P4HB*, and *SPHK1*) increased, the histological grade, pathological stage, T status, or N status of BLCA patients increased (*P* < 0.05; Fig. 5E–U). Furthermore, low-expression of *APOL1* and increased risk score were observed in patients with age > 65 (*P* < 0.05; Fig. 5A–Q). Survival analysis showed that high expression of the protective gene *APOL1* indicated a good prognosis (*P* < 0.01; Fig. 6A), while increased expression of risk gene *P4HB* resulted in a poor prognosis (*P* < 0.05; Fig. 6D). The other three risk genes (*DIRAS3*, *NAMPT*, and *SPHK1*) had no significant effect on survival outcome (*P* > 0.05; Fig. 6B, C, and E).

**Table 2:** Clinical utility of prognostic model related factors [*t*-value (*P* value)].

Genes	Age	Gender	Grade	Stage	T	M	N
*APOL1*	2.602 (0.011)	− 1.622 (0.109)	− 1.66 (0.115)	2.652 (0.010)	1.855 (0.068)	2.641 (0.031)	3.284 (0.001)
*DIRAS3*	− 1.412 (0.160)	0.985 (0.331)	3.272 (0.002)	− 2.918 (0.004)	− 2.442 (0.016)	1.283 (0.234)	− 1.72 (0.091)
*NAMPT*	− 0.159 (0.874)	1.112 (0.272)	4.022 (2.157e−04)	− 3.063 (0.003)	− 2.526 (0.013)	0.812 (0.445)	− 1.121 (0.266)
*P4HB*	− 1.763 (0.081)	0.175 (0.862)	2.993 (0.008)	− 2.112 (0.037)	− 0.98 (0.329)	0.416 (0.691)	− 2.375 (0.020)
*SPHK1*	− 1.269 (0.206)	0.635 (0.529)	7.326 (1.635e−11)	− 3.207 (0.002)	− 3.547 (5.201e−04)	0.61 (0.552)	− 1.364 (0.177)
Risk score	− 2.244 (0.026)	1.6 (0.116)	6.294 (1.13e−07)	− 4.839 (3.471e−06)	− 3.201 (0.002)	0.363 (0.724)	− 3.001 (0.003)

### Correlation between risk score and TME

**Figure 7:**
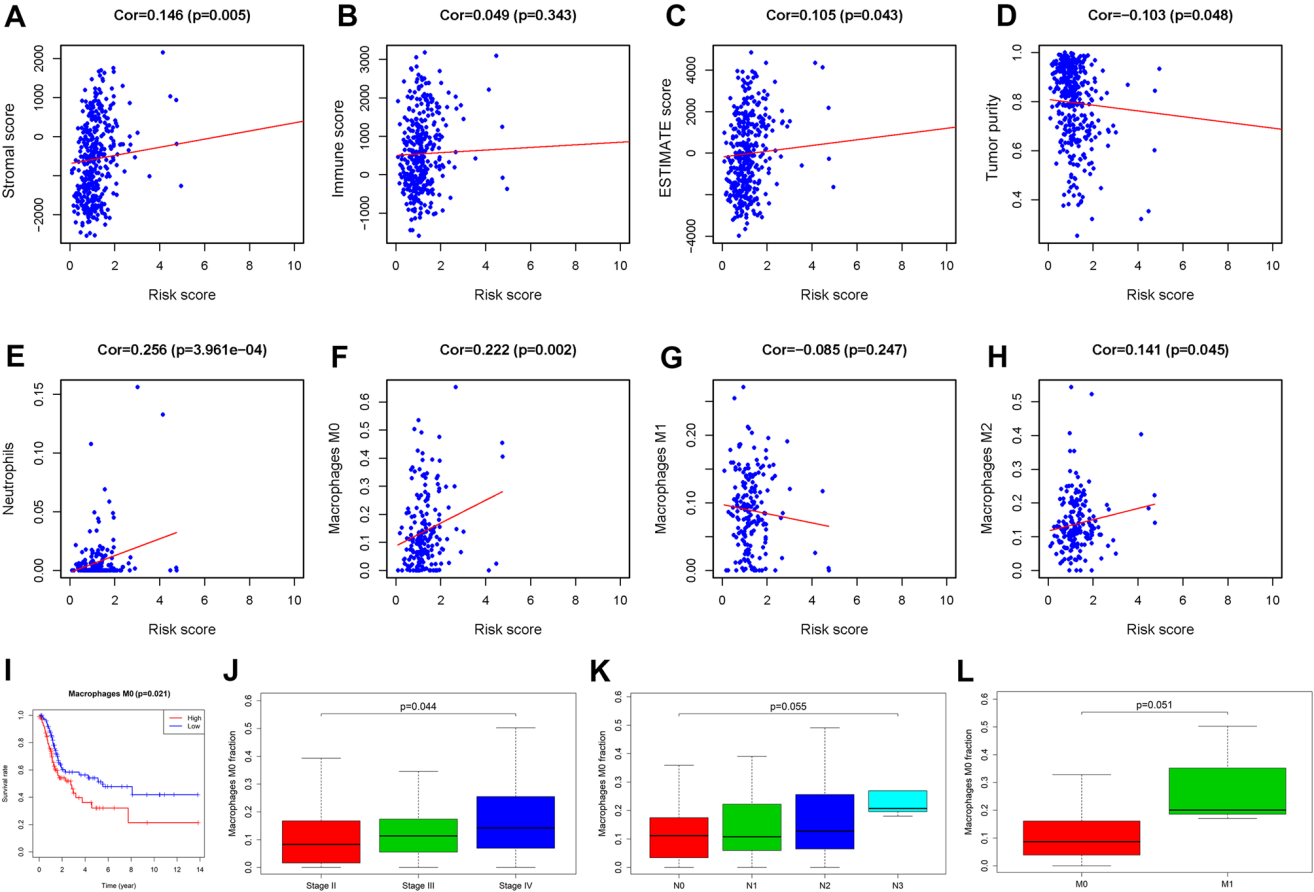
The correlation between risk score and TME. (**A**) Risk score and stromal score. (**B**) Risk score and immune score. (**C**) Risk score and ESTIMATE score. (**D**) Risk score and tumor purity. (**E**) Risk score and neutrophils. (**F**) Risk score and macrophage M0. (**G**) Risk score and macrophage M1. (**H**) Risk score and macrophage M2. (**I**–**L**) Macrophage M0 and clinical variables.

As shown in Fig. 7A–D, with the increase of risk score generated by present prognostic signature, tumor-infiltrating stromal cells (i.e., stromal score) and estimate score (sum of stromal score and immune score) significantly increased, meanwhile tumor purity significantly decreased (*P* < 0.05). Additionally, 22 types of tumor-infiltrating immune cells (TIICs) in TCGA-BLCA were analyzed with CIBERSORT, and the content of neutrophils, macrophages M0 and M2 increased with risk score (*P* < 0.05; Fig. 7E–H). Furthermore, a high proportion of macrophages M0 could lead to a poor prognosis, accompanied by increased pathological stage, N status, and M status of BLCA patients (*P* < 0.05; Fig. 7I–L), indicating that tumor-associated macrophage (TAM) might involve with PDEARGs mediated biological process.

### GO enrichment analysis

**Figure 8:**
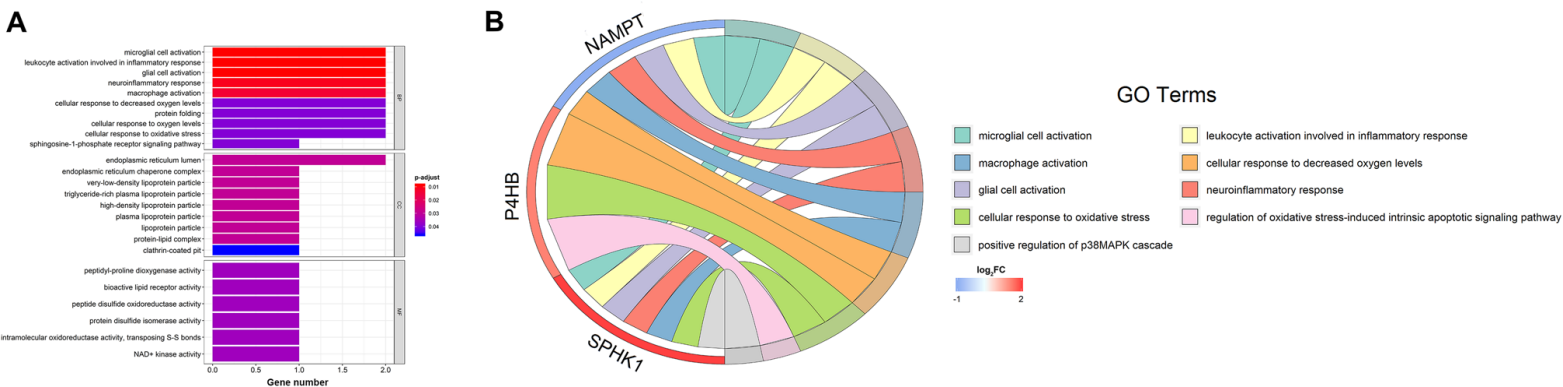
GO enrichment analysis of PDEARGs. (**A**) Bar plot of enriched GO terms. (**B**) Chord plot of enriched GO terms. FDR < 0.05 was considered statistically significant.

To further explore the relationship between PDEARGs and TAM, Gene Ontology (GO) enrichment analysis was employed. The five selected PDEARGs were associated with 52 biological processes (BPs), six molecular functions (MFs), and nine cellular components (CCs), among which immunologic process-related GO terms were significantly enriched, such as the activation of microglial cell, leukocyte, and macrophage as well as inflammatory response. Besides, oxidative stress and relevant apoptotic signal pathway were significantly enriched (FDR < 0.05; Fig. 8A). Therefore, TAM might be regulated by PDEARGs. To better explain the relationship between PDEARGs and GO terms, a chord plot was constructed, and three essential genes (*SPHK1*, *P4HB,* and *NAMPT*) that might participate in macrophage regulation were found (Fig. 8B).

### Construction of PDEARGs-based systematic signature

To promote clinical application and predictive accuracy of the model mentioned above, we constructed a systematic prognostic signature by integrating seven clinical variables and the five PDEARGs-based signatures. The clinical variables (i.e., age, histological grade, and TNM status) correlated highly with the aforementioned risk score were deleted to avoid overfitting. Then, the regression coefficients were used to weight selected variables, and the systematic risk of each patient was calculated with the following computational formula (Table 3):

$$\begin{aligned} {\text{Systematic}}\;{\text{risk}} & = \left( { - 0.5755 \times {\text{gender}}} \right) + \left( {0.6253 \times {\text{pathological}}\;{\text{stage}}} \right) \\ & \quad + \left( {0.5276 \times {\text{risk}}\;{\text{score}}\;{\text{generated}}\;{\text{from}}\;{\text{aforementioned}}\;{\text{model}}} \right);\;{\text{gender:female}} = 1,\;{\text{male}} = 0. \\ \end{aligned}$$

**Table 3:** The construction of PDEARGs-based systematic prognostic signature.

Variables	Coefficient	HR	HR.95L	HR.95H	*P* value
Gender	− 0.5755	0.5624	0.3085	1.0253	0.0603
Stage	0.6253	1.8687	1.2486	2.7968	0.0024
Risk score	0.5276	1.6949	1.2194	2.3558	0.0017

*HR* hazard ratio, *HR.95H and HR.95L* 95% confidence interval.

**Figure 9:**
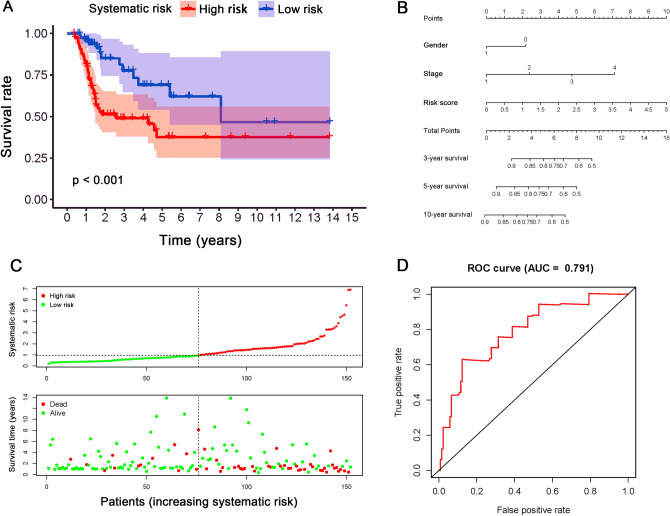
The construction and validation of PDEARGs-based systematic prognostic signature. (**A**) Survival analysis between two groups with different systematic risk. The 95% confidence interval was shown as a light-colored background around the Kaplan–Meier curve. (**B**) Nomogram of the systematic prognostic signature. (**C**) Risk plot encompassing the distribution of systematic risk and survival status of each patient. (**D**) ROC curve analysis of systematic risk.

The median of the systematic risk was 0.96, and with the increase of systemic risk, a bad prognosis of BLCA patients was observed (*P* < 0.001; Fig. 9A, C). Precisely, the three-year and 5-year OS rate was 49.0% and 37.6% in the high-risk group, while it was 77.6% and 69.0% in the low-risk group, respectively. A nomogram was constructed accordingly, and the AUC value for systematic prognostic signature was 0.791, which was more accurate than the pure PDEARGs-based model (Fig. 9B, D).

### External validation of both signatures

**Figure 10:**
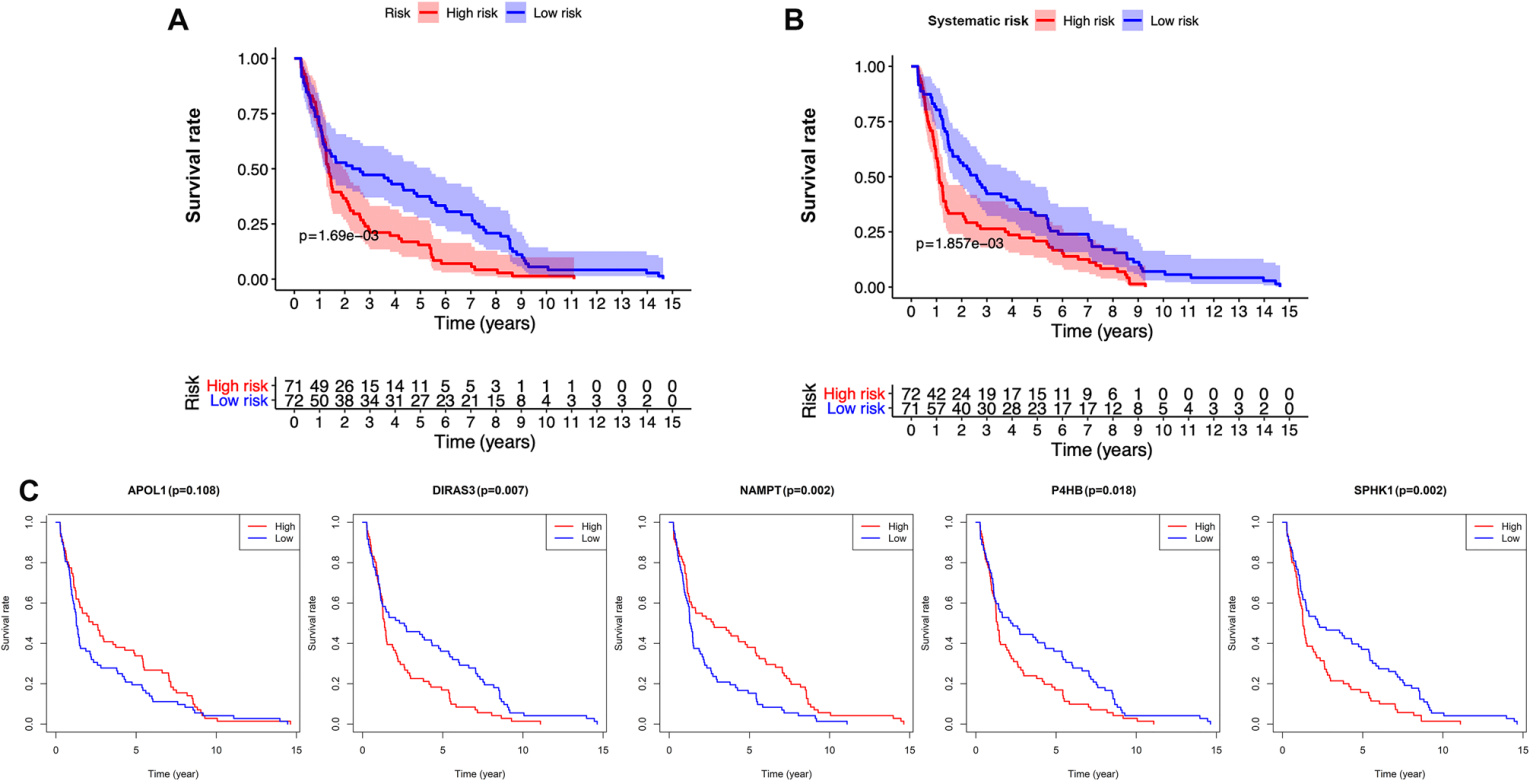
External validation of both signatures. (**A**) Validation of PDEARGs-based signature. (**B**) Validation of systematic signature. The 95% confidence interval was shown as a light-colored background around the Kaplan–Meier curve. (**C**) Survival analysis of five selected PDEARGs in external cohorts. The median expression level was used as the cut-off value.

Both PDEARGs-based prognostic signature and systematic signature were validated in external datasets. As shown in Fig. 10A and B, a significantly different survival outcome was observed between the two risk groups (*P* < 0.005, log-rank test). The OS rate at 5-year was 15.49% and 37.50% for PDEARGs-based high-risk and low-risk groups. The corresponding rate for systematic signature was 20.83% and 32.39%, indicating that both signatures could precisely predict BLCA prognosis regardless of internal and external cohorts. Besides, the prognostic value of five selected PDEARGs was estimated through survival analysis. Except for *APOL1,* the other four risk genes could effectively separate BLCA patients with different survival outcomes (*P* < 0.05, Fig. 10C). Though these results were not exactly consistent with Fig. 6, the general trend of survival curve between the high expressive and low expressive groups was similar.

## Discussion

BLCA is a frequent malignant disease with rising incidence and high recurrence rates^19^. Although new diagnostic and therapeutic strategies have been carried out over the past several decades, the clinical outcome of BLCA patients remains unsatisfied^4,20^. Hence, there is an urgent need to develop better diagnostic methods to accurately identify asymptomatic and recurrent individuals at an early stage and propose novel treatment targets to improve prognosis. Previous studies have revealed that autophagy plays an essential role in TME regulation, resulting in tumor cell migration and invasion, tumor stem cell maintenance, and therapy resistance^21,22^. Therefore, ARGs may be an ideal biomarker or indicator to predict the progression and prognosis of BLCA.

Recently, many malignant tumor-related studies have shown that ARGs-based signature or multigene expression patterns can favorably predict cancer prognosis^9,10,12^. For example, P4HB retrieved from HADb website has been identified as a prognostic biomarker for BLCA, which could dramatically inhibit the invasion and proliferation of bladder cancer cells, providing novel insights for autophagy-mediated tumor progression^23^. However, whether multigene-based prognostic signature can predict the outcome of BLCA patients aroused our interest. In present study, we identified five optimal PDEARGs based on a comprehensive analysis, among which *APOL1* was a protective gene, and the other four PDEARGs were risk genes. Then, we constructed a reliable PDEARGs-based prognostic model that could stratify TCGA-BLCA patients into two risk groups with statistically different survival outcomes. The risk score generated from the present signature could serve as an independent prognostic factor to predict patients’ OS, and the predictive accuracy (AUC = 0.724) was much better than other clinical parameters. As reported, AUC > 0.60 was regarded as acceptable for predictions^24^; thus, the present PDEARGs-based signature can precisely predict the prognosis of BLCA patients.

Subsequently, we analyzed the expression of the five PDEARGs in two risk groups and evaluated the relationship between present signature and certain clinical variables. Firstly, the expression of the protective gene *APOL1* was downregulated in the high-risk group, and the other four risk genes were upregulated in the patient with an increased risk score. Consistently, the RNA level of five PDEARGs detected through qRT-PCR obtained similar results as RNA-Seq data, indicating its potential for clinical transformation. However, we didn’t include a large sample in the present proof of concept study, which need further confirmation with prospective clinical study. Besides, *APOL1* expression significantly decreased with the increase of pathological stage, N and M status. The high expression of risk genes was accompanied by bad histological grade, pathological stage, TNM status, and prognosis. Based on the above, the present signature constructed with low-expression of protective gene and high-expression of risk genes could accurately predict BLCA patients' progression, including histological grade, pathological stage, TNM status, and survival outcome. What’s more, BLCA incidence increased with age^25^, and we found that *APOL1* level was down-regulated and risk score increased in elderly patients (age > 65), which further confirmed the reliability and clinical consistency of present model.

TME, which comprises recruited stromal cells and TIICs, has emerged as an important player in tumor progression, with the potential to be used in future treatment and diagnosis^26^. In present study, the TME of BLCA patients was analyzed with the ESTIMATE and CIBERSORT R package. With the increase of risk score generated from PDEARGs-based signature, tumor purity significantly decreased, and the proportion of stromal cells, neutrophils, and macrophage (M0 and M2 phenotypes) in tumor tissues significantly increased, which was correlated with bad pathological stage, detrimental TNM status, and poor prognosis. As reported, tumor-associated stromal cells can synthesize and secrete many pro-tumorigenic factors to promote cancer initiation, angiogenesis, invasion, and metastasis^27^. Additionally, emerging evidence indicates that elevated neutrophils are associated with detrimental outcomes in several solid tumors, and new strategies to decrease their presence and activity have shown efficacy in preclinical models^28,29^. TAM is another prominent component of TME, and the accumulation of M0 and M2 macrophages in tumors is most strongly associated with poor clinical outcomes^18^. Therefore, the ability to reflect the tumor-promoting microenvironment may explain why the present PDEARGs-based signature could precisely predict the prognosis of BLCA patients.

To explore the underlying mechanism by which the present prognostic model effectively stratified BLCA patients, GO enrichment analysis of the five PDEARGs was performed. As a result, several immune-related GO terms were significantly enriched, such as macrophage activation, inflammatory response, cellular response to decreased oxygen levels, and related apoptotic signaling pathway. It has been reported that ARGs are tightly related to hypoxia and hypoxia-induced metabolic reprogramming, among which *P4HB* is a crucial molecule that has been extensively studied^7,30^. The expression of *P4HB* significantly increased in several solid tumors, including bladder cancer^31^, kidney cancer^32^ and prostate cancer^30^, which is consistent with the present study. Besides, hypoxia and oxygen stress-induced autophagy and apoptosis could be regulated by ARGs such as *SPHK1*, *P4HB,* and *NAMPT*^33^. During the process mentioned above, inflammatory response would be initiated, and TME could be changed to recruit and activate macrophages. *NAMPT* is regarded as a pleiotropic modulator governing monocyte/macrophage differentiation, polarization, and migration^34^. Therefore, we speculate that the process of “PDEARGs → autophagy → TME change → TAM recruitment and polarization → tumor progression” would provide insights for present prognostic signature and propose potential treatment targets for BLCA patients.

Though the present model exhibits a promising value in predicting BLCA patients' prognosis, we intend to integrate clinical variables and risk scores generated from the model above to construct a systematic signature with promoted clinical utility and predictive accuracy. Notably, the novel systematic signature could more precisely predict the prognosis of TCGA-BLCA patients (AUC = 0.791). As reported, AUC > 0.75 was deemed to have an excellent predictive value^24^. The reliability of both PDEARGs-based signatures was also validated through external cohorts downloaded from the GEO database. A nomogram of the systematic signature was then prepared for clinicians to identify BLCA patients who need invasive therapy.

In conclusion, we constructed a valuable PDEARGs-based prognostic model that can precisely predict the prognosis of BLCA patients. This model's risk score can serve as an independent prognostic factor and can effectively reflect the tumor-promoting microenvironment. GO enrichment analysis revealed the underlying mechanism, which may provide insights for the present model and propose potential treatment targets for BLCA patients. Additionally, a systematic signature integrating clinical variables and the aforementioned five PDEARGs-based model was constructed for clinical application. Inevitably, large-scale, multi-center studies are necessary to confirm the clinical benefit of our results. In vitro or in vivo experiments need to be performed to provide more evidence for PDEARGs-based regulatory mechanism.

## Supplementary Information

Supplementary Table S1.

Supplementary Table S2.

## References

[1] Bray F (2018). Global cancer statistics 2018: GLOBOCAN estimates of incidence and mortality worldwide for 36 cancers in 185 countries. CA Cancer J. Clin..

[2] Lisio MA, Fu L, Goyeneche A, Gao ZH, Telleria C (2019). High-Grade serous ovarian cancer: Basic sciences clinical and therapeutic standpoints. Int. J. Mol. Sci..

[3] Powles T (2014). MPDL3280A (anti-PD-L1) treatment leads to clinical activity in metastatic bladder cancer. Nature.

[4] Butt SUR, Malik L (2018). Role of immunotherapy in bladder cancer: Past, present and future. Cancer Chemother. Pharmacol..

[5] He A (2019). Prognostic value of long non-coding RNA signatures in bladder cancer. Aging (Albany NY).

[6] Qiu H (2020). Identification and validation of an individualized prognostic signature of bladder cancer based on seven immune related genes. Front. Genet..

[7] White E, Mehnert JM, Chan CS (2015). Autophagy, metabolism, and cancer. Clin. Cancer Res..

[8] Levy J, Towers CG, Thorburn A (2017). Targeting autophagy in cancer. Nat. Rev. Cancer.

[9] Zhou Z (2019). Development and validation of an autophagy score signature for the prediction of post-operative survival in colorectal cancer. Front. Oncol..

[10] Liu Y (2019). Prognostic implications of autophagy-associated gene signatures in non-small cell lung cancer. Aging (Albany NY).

[11] Wang Z (2019). Development and validation of a nomogram with an autophagy-related gene signature for predicting survival in patients with glioblastoma. Aging (Albany NY).

[12] Gu Y (2016). Autophagy-related prognostic signature for breast cancer. Mol. Carcinog..

[13] Yue C, Ma H, Zhou Y (2019). Identification of prognostic gene signature associated with microenvironment of lung adenocarcinoma. PeerJ.

[14] Wan B, Liu B, Huang Y, Yu G, Lv C (2019). Prognostic value of immune-related genes in clear cell renal cell carcinoma. Aging (Albany NY).

[15] Yoshihara K (2013). Inferring tumour purity and stromal and immune cell admixture from expression data. Nat. Commun..

[16] Newman AM (2015). Robust enumeration of cell subsets from tissue expression profiles. Nat. Methods.

[17] Zeng D (2018). Gene expression profiles for a prognostic immunoscore in gastric cancer. Br. J. Surg..

[18] Ali HR, Chlon L, Pharoah PD, Markowetz F, Caldas C (2016). Patterns of immune infiltration in breast cancer and their clinical implications: A gene-expression-based retrospective study. PLoS Med..

[19] Hu J (2019). The identification of new biomarkers for bladder cancer: A study based on TCGA and GEO datasets. J. Cell. Physiol..

[20] Berdik C (2017). Unlocking bladder cancer. Nature.

[21] Monkkonen T, Debnath J (2018). Inflammatory signaling cascades and autophagy in cancer. Autophagy.

[22] Mowers EE, Sharifi MN, Macleod KF (2018). Functions of autophagy in the tumor microenvironment and cancer metastasis. Febs J..

[23] Lyu L (2020). Significant prognostic value of the autophagy-related gene P4HB in bladder urothelial carcinoma. Front. Oncol..

[24] Cho SH (2019). The AP2M1 gene expression is a promising biomarker for predicting survival of patients with hepatocellular carcinoma. J. Cell. Biochem..

[25] Babjuk M (2018). Bladder cancer in the elderly. Eur. Urol..

[26] Roma-Rodrigues C, Mendes R, Baptista PV, Fernandes AR (2019). Targeting tumor microenvironment for cancer therapy. Int. J. Mol. Sci..

[27] Bussard KM, Mutkus L, Stumpf K, Gomez-Manzano C, Marini FC (2016). Tumor-associated stromal cells as key contributors to the tumor microenvironment. Breast Cancer Res..

[28] Ocana A, Nieto-Jiménez C, Pandiella A, Templeton AJ (2017). Neutrophils in cancer: Prognostic role and therapeutic strategies. Mol. Cancer.

[29] Coffelt SB, Wellenstein MD, de Visser KE (2016). Neutrophils in cancer: Neutral no more. Nat. Rev. Cancer.

[30] Xie L (2020). Autophagy-related gene P4HB: A novel diagnosis and prognosis marker for kidney renal clear cell carcinoma. Aging (Albany NY).

[31] Sanchez-Carbayo M, Socci ND, Lozano J, Saint F, Cordon-Cardo C (2006). Defining molecular profiles of poor outcome in patients with invasive bladder cancer using oligonucleotide microarrays. J. Clin. Oncol..

[32] Yusenko MV (2009). High-resolution DNA copy number and gene expression analyses distinguish chromophobe renal cell carcinomas and renal oncocytomas. BMC Cancer.

[33] Liu H, Ma Y, He HW, Zhao WL, Shao RG (2017). SPHK1 (sphingosine kinase 1) induces epithelial-mesenchymal transition by promoting the autophagy-linked lysosomal degradation of CDH1/E-cadherin in hepatoma cells. Autophagy.

[34] Travelli C, Colombo G, Mola S, Genazzani AA, Porta C (2018). NAMPT: A pleiotropic modulator of monocytes and macrophages. Pharmacol. Res..

[35] Zhang, K. *et al*. Prognostic value and underlying mechanism of autophagy-related genes in bladder cancer. *Research Square (PREPRINT)* (2020). 10.1038/s41598-022-06334-0 PMC8828781 35140317

