# Supplementary material for: Prognostic value and underlying mechanism of autophagy-related genes in bladder cancer
Source: Sci Rep. 2022 Feb 9;12:2219. doi: 10.1038/s41598-022-06334-0 (PMC8828781; doi:10.1038/s41598-022-06334-0)
Supplement: Supplementary file 1 — Supplementary Table S1. [file 41598_2022_6334_MOESM1_ESM.docx]

**Supplementary Table 1.** Clinical characterization of selected patients (n=3).

|  | **Sex** | **Age** | **Clinical stage** | **Invasive stage** | **Pathological grade** | **Ki-67** |
| --- | --- | --- | --- | --- | --- | --- |
| **Patient 1** | Male | 77 | T4bN0M0 | Muscle | High grade | >15% |
| **Patient 2** | Male | 60 | T3aN0M0 | Muscle | Low grade | <15% |
| **Patient 3** | Male | 61 | T2bN1M0 | Non-muscle | High grade | >15% |
