# Supplementary material for: Prognostic value and underlying mechanism of autophagy-related genes in bladder cancer
Source: Sci Rep. 2022 Feb 9;12:2219. doi: 10.1038/s41598-022-06334-0 (PMC8828781; doi:10.1038/s41598-022-06334-0)
Supplement: Supplementary file 2 — Supplementary Table S2. [file 41598_2022_6334_MOESM2_ESM.docx]

**Supplementary Table 2.** Raw data from qRT‑PCR.

| **Gene** | **Patients** | **Normal** | | | **Tumor** | | |
| --- | --- | --- | --- | --- | --- | --- | --- |
| ***APOL1*** | 1 | 1.0090 | 0.9779 | 1.0135 | 0.1719 | 0.1575 | 0.1638 |
|  | 2 | 0.8370 | 1.0737 | 1.1127 | 0.0323 | 0.0504 | 0.0420 |
|  | 3 | 1.1101 | 0.9767 | 0.9223 | 0.0410 | 0.0420 | 0.0452 |
| ***DIRAS3*** | 1 | 0.3120 | 0.4093 | 0.6409 | 0.9951 | 0.8763 | 1.1469 |
|  | 2 | 0.1294 | 0.3894 | 0.2601 | 1.1128 | 1.5858 | 1.5341 |
|  | 3 | 0.6759 | 0.3446 | 0.5749 | 1.1401 | 0.7647 | 1.1470 |
| ***NAMPT*** | 1 | 0.5640 | 0.5084 | 0.7887 | 0.9658 | 1.0884 | 0.9514 |
|  | 2 | 0.5004 | 0.6942 | 0.4735 | 1.0628 | 0.9489 | 0.9916 |
|  | 3 | 0.6127 | 0.4373 | 0.5106 | 0.8705 | 1.0076 | 1.1400 |
| ***P4HB*** | 1 | 0.5686 | 0.6579 | 0.6112 | 0.8979 | 1.0739 | 1.0371 |
|  | 2 | 0.4867 | 0.4926 | 0.3767 | 0.9317 | 1.0667 | 1.0062 |
|  | 3 | 0.3892 | 0.3539 | 0.3801 | 1.0570 | 1.0137 | 0.9333 |
| ***SPHK1*** | 1 | 0.3861 | 0.2247 | 0.3858 | 1.6176 | 1.4026 | 1.5356 |
|  | 2 | 0.3567 | 0.2569 | 0.1620 | 1.8942 | 1.6160 | 1.6920 |
|  | 3 | 0.2154 | 0.2457 | 0.2845 | 1.2640 | 0.9564 | 0.8272 |
